# Application of pharmacogenetics in oncology

**DOI:** 10.1186/s40364-020-00213-4

**Published:** 2020-08-17

**Authors:** Nelly N. Miteva-Marcheva, Hristo Y. Ivanov, Dimitar K. Dimitrov, Vili K. Stoyanova

**Affiliations:** 1 Department of Pediatrics and Medical Genetics, Medical University Plovdiv, Plovdiv, Bulgaria; 2 Department of Medical Genetics, University Hospital “St. George” Plovdiv, Plovdiv, Bulgaria

**Keywords:** Pharmacogenetics, Pharmacogenomics, Oncology, Pharmacogenetic biomarkers

## Abstract

The term “pharmacogenetics” is used to describe the study of variability in drug response due to heredity. It is associated with “gene – drug interactions”. Later on, the term “pharmacogenomics” has been introduced and it comprises all genes in the genome that can define drug response. The application of pharmacogenetics in oncology is of a great significance because of the narrow therapeutic index of chemotherapeutic drugs and the risk for life-threatening adverse effects. Many cancer genomics studies have been focused on the acquired, somatic mutations; however, increasing evidence shows that inherited germline genetic variations play a key role in cancer risk and treatment outcome. The aim of this review is to summarize the state of pharmacogenomics in oncology, focusing only on germline mutations. Genetic polymorphisms can be found in the genes that code for the metabolic enzymes and cellular targets for most of the chemotherapy drugs. Nevertheless, predicting treatment outcome is still not possible for the majority of regimens. In this review, we discuss the most comprehensively studied drug-gene pairs – present knowledge and current limitations. However, further studies in larger groups of cancer patients are necessary to be conducted with precise validation of pharmacogenetic biomarkers before these markers could be routinely applied in clinical diagnosis and treatment.

## Introduction

Treatment in oncology has made a great progress over the past decade due to the recent revolution in medical interventions. Narrow therapeutic indices, variable overall response rates and clinical outcomes, and toxicities from chemotherapies are examples of the problems that arise from cancer treatment [1]. Although many patients have similar clinical presentation, they manifest quite different response to one and the same treatment. Some of the therapeutic schemes appear to be ineffective in patients with cancer. On the other hand, this may lead to adverse drug reactions or might increase the likelihood of overtreatment. Optimizing treatment regimens for the individual patient will conceivably lead to better clinical outcomes [1]. Over the recent years, owing to the advancement of medical genomics and proteomics we have improved our knowledge of personal differences in pharmacokinetics and pharmacodynamics based on genetic makeup.

Pharmacogenetics is the study of the genetic factors that influence drug response and toxicity. It focuses on variation within the human genome. While somatic mutations are associated with molecular markers found in the tumour tissue, pharmacogenomics studies the genetic markers that have predictive value of outcome from pharmacologic treatment [2, 3].

More thorough knowledge and common application of pharmacogenomic markers is particularly important and desirable in oncology practice, as the therapeutic index of the drugs is usually quite narrow, and the consequences of adverse effects and toxicity might be severe or even life-threatening. If it is possible to predict which individuals can benefit from treatment and which ones may suffer from chemotherapy-related toxicity, then the overall care of cancer patients would be considerably improved.

The main purpose of this review is to revise the state of pharmacogenetics nowadays in the field of oncology. We are going to focus especially on germline genetic variations related to oncologic therapeutics. We will also consider the limitations and the means by which pharmacogenomics can be implemented in the routine clinical practice for oncology patients.

## Major current pharmacogenetic data in oncology

The current number of well-known and studied drug-gene pharmacogenomic pairs in oncology is relatively negligible compared to other drugs. Examples of these pairs will be discussed below.

### Dihydropyrimidine dehydrogenase genotype and fluoropyrimidine dosing

Fluoropyramidines, such as 5-Fluorouracil (5-FU), capecitabine and tegafur, are widely used in the treatment of many cancers, such as colorectal carcinoma, head-neck cancer and breast cancer. Over the past decades, increased understanding of the mechanism of action of the drug has led to the development of new methods that can augment its activity. In spite of the recent progress, drug resistance remains an important limitation to the application of 5-FU.

Dihydropyramidine dehydrogenase (DPD) participates in the 5-FU metabolism by converting up to 80% into inactive metabolites and thus it is responsible for its elimination. It is encoded by the DPYD gene. Because of the great variety for DPD between individuals, different effects from treatment with 5-FU has been observed – efficacy, resistance and toxicity. The most common side effects in patients with deficiency of DPD enzyme are myelosuppression, mucositis, neurotoxicity and diarrhea. Although more than 30 gene polymorphisms (SNPs, as well as insertions or deletions) are known in the DPYD gene, only 3 of them are related to low DPD activity and higher 5-FU toxicity.

In 1984 it has been suggested that DPD deficiency is connected with an excess of thymine and uracil [4]. There are some studies confirming this postulation. In a clinical case of a 27-year-old woman, who was treated with 5-FU, severe neurological and haematological adverse effects were observed. In her urinalysis there were high levels of thymidine and uracil [4]. In another case an autosomal recessive pattern of inheritance was discussed: when administrating a test dose of 5-FU both to the patient and their father, there was prolonged elimination half time without any catabolites in the patient, while in their father’s sample partial DPD deficiency was observed [4]. There are numerous variants that have been found after DPYD gene sequencing [4]. The most significant one is G to A polymorphism in intron 14 (inv14 + 1G > A or DPYD*2A; exon skipping mutation). In a study of oncology patients treated with 5-FU, about 55% of those who were DPD deficient developed grade 4 neutropenia, while only 13% of the patients with normal DPD activity suffered this adverse reaction. However, this is not the only polymorphism that has been discovered so far. Multiple other variants have been described: IVS11 + 1G > T, 731A > C (E244V), 1651G > A (A551T), G1601A (DPYD*4), T1679G(DPYD*13) (see also Table 1). All these variations lead to decreased activity of DPD. Yet not all of the cases with decreased DPD activity can be explained with DPYD polymorphisms. In summary, genetic testing for DPYD*2A as a predictive marker of 5-FU toxicity has a positive predictive value of about 50%, compared to about 95% negative [5]. Furthermore, 5-FU toxicity cannot only be due to DPD deficiency. Several other genes may have an effect on 5-FU activity: ABCB1, methylene tetrahydrofolate reductase, and thymidylate synthetase [11]. However, there are not enough studies for them, so the results so far seem to be contradictory.

**Table 1:** Summary of the most common enzymes/transport systems, the genes that code for them, metabolised drugs and expected therapy outcomes

Enzyme/ Transporter	Gene	Variant alleles	Drug molecule	Effect	Neoplasm	Reference
**Dihydropyramidine dehydrogenase**	DPYD	DYPD*13, DPYD*8, DPYD*7, DPYD*12, DPYD*3, DYPD*4, DPYD*2A, DPYD*9A	Fluoropyramidines – 5-fluorouracil, capecitabine, tegafur	Efficacy/Resistance/Toxicity	colorectal carcinoma, breast cancer	[4]
					head-neck cancer	[5]
**Thymidylate synthetase**	TYMS	TSER*2, TSER*3	5-fluorouracil, capecitabine	Efficacy/Toxicity	colorectal, bladder, gastric carcinoma	[4]
**Methylene tetrahydrofolate reductase**	MTHFR	667 C > T, 1298A > C,	5-fluorouracil, methotrexate	Efficacy/Toxicity	colorectal carcinoma, ovarian cancer, gastric cancer	[4]
**Thiopurine S-methyltransferase**	TPMT	TPMT*2–24 (TPMT*1, TPMT*2, TPMT*3A, TPMT*3B, TPMT*3C, TPMT*4)	azathioprine, mercaptopirune, thioguanine	Dosage/Toxicity/ADR	acute lymphatic leukemia	[4]
					ovarian cancer	[6]
					breast cancer	[7]
**Uridine diphosphate glucuronosyl transferase**	UGT1A1	UGT1A1*27, UGT1A1*28, UGT1A1*6	irinotecan	Toxicity/ADR	colorectal carcinoma	[4]
**Glutathione S-transferases**	GST	GSTM1, GSTP1	platinum compounds - cyclofosfamide, carboplatin, doxorubicin, cisplatin, oxaliplatin	Efficacy/Toxicity/ADR	colorectal cancer	[5]
					bladder, head and neck, lung, ovarian and testicular cancer	[7]
**Excision repair cross complementing group 1**	ERCC1	496 C > T, 8092 C > A, 19007 T > C	platinum containing anti-cancer drugs	Efficacy/Toxicity	non-small cell lung carcinoma, bladder cancer	[4]
					colorectal carcinoma	[8]
**Excision repair cross complementing group 2**	ERCC2	965 G > A, 225 A > C	platinum containing anticancer drugs - oxaliplatin	Efficacy/Toxicity	colorectal cancer	[4, 8]
					ovarian cancer	[9]
					non-small cell lung carcinoma	[10]
**ATP binding cassettes**	ABCB1, ABCC2, ABCG2	1236 C > T, 3435 C > T, 2677 G > T, 421 C > A, ABCC2*2	irinotecan	Efficacy/Toxicity/Resistance	ovarian cancer	[10]
**X-ray cross complementing group 1**	XRCC1	1301 G > A	platinum anti-cancer drugs, 5-fluorouracil	Efficacy/Toxicity	colorectal, gastric and non-small cell lung carcinoma	[4]
**Cytochrome P450 2D6**	CYP2D6	about 80 CYP2D6 variant alleles; most common ones - CYP2D6*4, CYP2D6*3, CYP2D6*5 and CYP2D6*6	tamoxifen	Efficacy/Toxicity	breast cancer	[4]

### Thymidylate synthetase (TS)

DPD is not the only enzyme responsible for 5-FU metabolism. Another one that is involved in the drug’s pathway is thymidylate synthetase. TS is associated with thymidine synthesis and it is a target for 5-FU which inhibits it. The gene encoding for TS is TYMS. Two different alleles have been discovered for this gene – with a 2-repeat sequence (TSER*2) and with a 3-repeat sequence (TSER*3) (see also Table 1). TSER*3 is associated with better outcome in patients with colorectal carcinoma when treated with 5-FU, compared to individuals with TSER*2 polymorphism [4]. Nevertheless, not all of the patients with TSER*3 allele appear to have a worse outcome. The most probable explanation is another variation (a G > C SNP), which leads to lower TS activity, comparable with the TSER*2 allele. This SNP is discovered in about 29–57% of TSER*3 alleles [4]. TS amplifications is used as a prognostic factor for treatment with 5-FU not only in colorectal carcinoma, but also in bladder and gastric carcinoma [12]. Applying TYMS together with DPYD genotyping may contribute to choosing patients who will have a better response on 5-FU therapy and less adverse effects [4].

### Methylene tetrahydrofolate reductase (MTHFR)

Methylene tetrahydrofolate reductase is an enzyme which participates in 5-FU and methotrexate (MTX) pathway. It plays an important role in the metabolism of folate and methionine and thus in the synthesis and methylation of DNA. MTHFR metabolizes a 5-FU substrate (5,10 methylene tetrahydrofolate), and therefore decreased function of this enzyme is associated with enhanced 5-FU activity, while MTX sensitivity is reduced [4]. The most frequent polymorphism for the gene that codes for MTHFR is 677C > T (see also Table 1). The MTHFR 677C > T variant was first discovered by Frosst et al., as being the causal variant for the thermolabile MTHFR protein. The thermolabile MTHFR protein is associated with 50% lower activity in vitro. It was also the first genetic risk factor identified for spina bifida. There is a huge body of work on this variant, in association with a variety of drugs, phenotypes and diseases, and much of it is contradictory. It has been examined in a myriad of diseases including cardiovascular diseases, cancers, disorders of pregnancy and development and in the context of drugs such as methotrexate (both as chemotherapy and for inflammation). Furthermore, studies have found that the T allele is associated with higher total homocysteine than the C allele particularly in individuals with lower plasma folate. In studies of methotrexate-treated pediatric acute lymphoblastic leukemia patients, the T allele was associated with a lower probability of event free survival but was not a risk factor for toxicity or seizures. Some studies have shown that the T allele may be protective compared to the C allele in disease incidence (colorectal neoplasms, breast neoplasms). Larger studies representing different populations are needed to determine the role of this polymorphism in response to antifolates and antimetabolites and to conclusively define its role in disease.

In one study of 43 patients with ovarian cancer, treated with MTX, grade 3–4 toxicity was observed in 77% of those who were homozygous for the TT genotype, 6% in the heterozygous ones and 8% for the individuals with CC homozygous genotype [4]. The levels of homocysteine in those patients who were homozygous for the TT genotype were higher and were associated with increased toxicity. Furthermore, patients with TT genotype showed better outcome when treated with 5-FU. In a clinical trial of 43 people with colorectal carcinoma who took 5-FU, the homozygous for TT genotype presented with better response and overall survival [4, 13]. Although many different studies have been carried out so far concerning the 5-FU metabolism and the significance of the DPYD, TYMS and MTHFR gene mutations, it is yet not possible to predict 5-FU toxicity. Consequently, further studies need to be conducted in order pharmacogenetics to become a general practice.

### Thiopurine S-methyltransferase (TPMT) and thiopurine dosing

Thiopurines are used not only for non-malignant diseases (IBD, rheumatoid arthritis), but they are also applicable for anticancer treatment in haematological malignancies [5]. The following three prodrugs azathioprine, mercaptopirune and thioguanine (TG) are inactivated by TPMT and produce the same active TG nucleotide (TGN) metabolites. It has been discovered that there is a correlation between TPMT activity and TGN concentrations. In patients who are homozygous for the inactive allele, severe myelosuppression is observed. Moderate to severe myelosuppression may be also seen in individuals who are heterozygous when normal thiopurine dosage is administered [5].

The anticancer drugs azathioprine, 6-mercaptopurine (6-MP) and 6-thioguanine are most often used for ALL treatment regimens [14, 15]. Their metabolism is regulated by TPMT. On the one hand, this enzyme inactivates the drugs through methylation and thus acts on their toxicity and, on the other hand, it converts 6-MP into methylthionosine 5-prime monophosphate (a metabolite which leads to inhibition of the de novo purine synthesis and may lead to toxic effects) [4, 16]. It is now known that due to genetic heterogeneity TPMT activity widely differs in a population [4]. Studies have found 3 different phenotypes: with normal, intermediate and absent TPMT activity respectively. There is an indisputable inverse correlation between decreased TPMT activity and 6-MP concentrations in red blood cells and ALL blasts. In order serious adverse effects to be avoided, it is necessary dose adjustments to be made depending on the TPMT activity [15]. Although the total number of TPMT variants that have been discovered is more than 20 (TPMT*2–24) [4, 17], the most common ones are TPMT*2 (found in an 8-year-old girl treated for ALL who developed severe haematological toxicity from conventional oral doses of 6-MP [4]) and TPMT*3A, both of which cause amino acid changes leading to breakdown of TPMT protein and thus decreased activity [4, 18] (see also Table 1). The frequency of TPMT variation in Caucasians is 10% with most common mutation TPMT*3A, while in Asian population the most widely seen mutation is TPMT*3C [4].

The most significant advantage of genotype testing of TPMT gene for the dosage assessment of thiopurine is that severe myelosuppression can be avoided without compromising on treatment efficacy. TPMT testing is widely available worldwide in routine service laboratories but it depends on the clinical specialty whether testing is implemented or not. While dermatologists have quickly adopted the routine TPMT testing, gastroenterologists do not specifically recommend TPMT screening. On the other hand, TPMT testing is obligatory prior to the use of mercaptopurine in childhood leukaemia. Furthermore, TPMT is cost effective – the cost of in-patient care for one TPMT deficient patient inadvertently treated with azathioprine has been estimated to cover the cost of over 400 tests for TPMT activity [19].

### Uridine diphosphate glucuronosyl transferase (UGT) genotype and irinotecan

UGT-1 is the main enzyme in the glucuronidation of bilirubin, as well as a lot of lipophilic drugs, such as the active metabolite of irinotecan – SN-38. Irinotecan is an anticancer drug, used in many therapeutic regiments for the treatment of colorectal carcinoma. Mutations in the UGT-1 gene can also cause Gilbert syndrome [20]. SN-38 is mainly cleared by the enzyme uridine diphosphate glucoronyltransferase 1A1 (UGT1A1). There are different variations of the gene but the most important one is UGT1A1*28 (an insertion of the element TA in the promoter region of the UGT1 gene) [4, 21]. These changes impair enzyme activity and affect irinotecan metabolism. In people who are homozygous for UGT1A1*28 allele, UGT1 activity is decreased by 70% [4]. Many different studies have showed that variations in the UGT1 gene are associated with increased toxicity (severe diarrhea and neutropenia) due to impaired irinotecan activity [4, 22]. The prevalence of the UGT1A1*28 allele differs between populations: highest in the African (45%) and lowest in the Asian (7–17%), while for the Caucasians the percentage is 22–39% [4, 23]. However, in Asian patients, except for this polymorphism, some other variants of the UGT1A1 gene were found – UGT1A1*6 and UGT1A1*27 alleles [24] (see also Table 1). Therefore, a population specific studies are necessary to be conducted in order clinicians to be able to tailor treatment with irinotecan.

### Glutathione S-transferases gene polymorphism and platinum compounds

Glutathione S-transferases (GSTs) comprise a family of enzymes responsible for the detoxification of xenobiotics including platinum compounds. There are several classes of GSTs, each of which is encoded by a different gene or gene family. Polymorphisms in the genes are supposed to result in an altered effect or toxicity manifestation when oncology patients are treated with drugs such as cyclofosfamide, carboplatin, doxorubicin, cisplatin.

GST is encoded by GSTP1. The most frequent polymorphism in this gene, which is associated with a better therapeutic outcome from treatment with oxaliplatin, is the non-synonymous SNP in exon 5 (313 A > G). It is detected in about 40–45% of the Caucasian population and in 27% of the Asian population [4]. One study of 107 patients with advanced CRC who were treated with 5-FU/oxaliplatin showed significant difference between individuals with the variant genotype and those with the wild genotype: the first group had a median survival of 24.9 months, while he second one – 7.9 months [4]. In a group of 64 patients with gastrointestinal cancer, who received oxaliplatin based therapy, the reduced activity of GST was associated with increased toxicity [4, 25]. Severe (grade 3) neuropathy has more often been seen in patients who are with the wild type genotype.

A published meta-analysis on glutathione S-transferase gene polymorphisms in patients with non-small cell lung cancer (NSCLC) revealed that both the null GSTM1 and the GG genotype of GSTP1 Ile105Val gene were associated with better clinical outcome and therapeutic response to cisplatin-based chemotherapy. The GSTP1 Ile105Val gene polymorphisms were more frequent in the East-Asian patients with NSCLC rather than the Caucasian ones. Chinese individuals with NSCLC showed better therapy response, which was associated with the fact that they were carriers of the null GSTM1 polymorphism. However, in order the role of the GST polymorphisms to be studied further, more thorough and ethnically diverse population clinical trials are necessary to be conducted in the future [26].

### Excision repair cross complementing group 1 (ERCC1)

The ERCC1 gene participates is the nucleotide excision pathway and is also connected with a gene specific repair which is realized by platinum containing anti-cancer drugs [27]. It has been found that high ERCC1 levels in patients with bladder cancer are associated with worse outcome [4, 28]. In non-small cell lung carcinoma, however, a polymorphism in exon 4 (496 C > T), is associated with better survival rate due to the decreased activity of ERCC1. In another clinical trial, including 91 patients with advanced CRC and treated with 5-FU/oxaliplatin, the above mentioned polymorphism was studied. The results showed that the patients who were homozygous for the variant type had a significantly higher response rate (61.9%), compared to those who were heterozygous (42.3%) or with the wild (21.4%) genotype [4, 29]. In summary, enhanced DNA repair leads to reduced efficacy of platinum based drugs. Still, more studies are necessary to be conducted in order to confirm this data for some contradictory results have also been published.

### Excision repair cross complementing group 2 (ERCC2)

Another gene which belongs to the nucleotide excision repair pathway is the ERCC2. A lot of single nucleotide polymorphisms have been found in this gene but the most common ones include 965 G > A (Asp321Asp) and 225 A > C (Lys751Gln) (see also Table 1) and they are associated with decreased DNA repair capability [4, 30]. In a group of 73 patients with advanced CRC who were treated with 5-FU/oxaliplatin, those who carried the 225 A > C polymorphism was associated with poorer clinical outcome and long term survival. The average survival for the patients with the wild type genotype was about 17.4 months, while it was 12.8 and 3.3 months for the heterozygous and the homozygous ones respectively [4].

Both ERCC1 and ERCC2 genes are important for the DNA repairing system and, therefore, are involved in the nucleotide excision repair pathway. A clinical study has been conducted in the Chinese population, in 2012 among 213 patients with the disease and 240 cancer-free controls suggested that SNPs in these genes are responsible for increased risk of CRC. Four functional SNPs were genotyped: ERCC1 Asn118Asn, C8092A, ERCC2 Asp312Asn, and Lys751Gln. It was found that individuals with ERCC1 C8092A polymorphism AA and CA/AA variant genotypes had significantly greater risk for colorectal carcinoma, compared with the CC genotype. Yet, no other SNPs were observed to have any important association with CRC. Thus, the ERCC1 C8092A polymorphism may become an important marker for colorectal cancer susceptibility amongst the Chinese population. However, larger studies are necessary to confirm these findings before their implementation in everyday practice [31].

### ATP-binding cassettes (ABCB1, ABCC2, ABCG2)

The ATP-binding cassette transporters (also called ABC transporters) represent a transport system superfamily that utilizes the energy of ATP binding and hydrolysis for the transport of different substrates across the cell membranes. Any change in their activity may lead to variability in the clearance of different drugs (including chemotherapeutic agents).

The ABCB1 gene (also called MDR1 – multi drug resistance) encodes for a P-glucoprotein. Overexpression of this glucoprotein is seen in cells that are resistant to specific anti-cancer regiments [32]. There are two synonymous SNPs (C1236T in exon 12 and C3435T in exon 26) and one non-synonymous SNP (G2677T in exon 2) that seem to be linked in MDR1*2 haplotype [4]. They have been seen in the European-American population (up to 62%), in the African-American population (13%), as well as in the Asian population. This haplotype leads to upregulation of P-glycoprotein, increased activity of the drug transporter and reduced SN-38 clearance [4].

Some other drug transporters involved in irinotecan metabolism are ABCG2 and ABCC2. The first one is also known as breast cancer resistance protein and a specific 421 C > A change is responsible for irinotecan disposition, while the second one has a ABCC2*2 haplotype that is associated with reduced adverse effects from irinotecan treatment (most commonly diarrhea) [33]. In a study of 167 patients on irinotecan therapy only 10% with haplotype ABCC2*2 had diarrhea compared to 44% in the other patients [4].

Current data implicate specific drug transporters, including the ATP superfamily, as a key factor for drug resistance and altered response. Recently, many different genetic polymorphisms in these transporters have been identified. Future studies can further evidence the role of genetic heterogeneity in ATP transporters and thus might allow for targeted and individualized therapy with minimal toxicity and maximal efficacy [34].

### X-ray cross complementing group 1 (XRCC1)

DNA repair protein XRCC1, which in humans in encoded by XRCC1 gene, is involved in the DNA single-strand breaks formed by exposure to ionizing radiation and alkylating agents, in the base excision repair and nucleotide excision repair. These mechanisms have an influence on the efficacy of platinum anti-cancer drugs. One SNP (1301 G > A; Arg399Gln) leads to mutated base excision capacity, higher risk for developing cancer and worse response in individuals with advanced CRC [35]. In a clinical trial of 61 patients with advanced CRC who received 5-FU/oxaliplatin therapy, 73% of people with a good response were carriers of the wild type genotype while none of the responders were homozygous [4]. It is also found that in NSCLC patients with this variant allele the survival rate was significantly shorter [4].

Molecular studies demonstrate the association between SNPs in XRCC1, the risk of different cancers (gastric cancer, for example) and their predictive value for treatment outcome [36]. In one study, comprising 612 gastric cancer patients, immunohistochemistry (IHC) was used to evaluate XRCC1 protein expression profiles on surgical specimens. It was found that XRCC1 IHC-negative patients benefited more than the IHC-positive ones from platinum-based adjuvant chemotherapy. These results support the idea that XRCC1 negative expression make tumours more sensitive to platinum-based chemotherapeutics. Detecting XRCC1 expression in people with gastric cancer can provide a clinical guidance when choosing the optimal adjuvant therapy. However, more large-scale studies are necessary to confirm the exact mechanisms [37].

### CYP2D6

One of the most commonly used drugs for hormone-dependent breast cancer treatment is tamoxifen. Breast cancers are hormone-dependent when they express oestrogen receptor (ER+) and thus oestrogen is usually necessary for their growth. Some medicines are called selective ER modulators as they inhibit oestrogen binding to the ERs and reduce or exclude oestrogen-driven proliferation of ER+ tumours. Tamoxifen is an example of selective ER modulator and is widely used for treatment of both premenopausal and postmenopausal women with metastatic breast cancer, for adjuvant chemotherapy and for preventive therapy for women with high risk of developing breast cancer. Its pharmacological activity depends on the hepatic enzyme cytochrome P450 2D6 (CYP2D6), which converts tamoxifen to its active metabolite endoxifen. It is observed that patients with reduced CYP2D6 activity either as a result of their genotype, or because of intake of other medicines, inhibiting CYP2D6, produce less endoxifen, which, on the other hand, leads to inferior therapeutic benefit [4].

To date, about 80 CYP2D6 variant alleles have been found, but the most common ones include CYP2D6*4, CYP2D6*3, CYP2D6*5 and CYP2D6*6 [38] (see also Table 1). Many polymorphisms are “silent” and result in alleles which express proteins with normal CYP2D6 activity (called extensive metabolizer (EM) alleles). Other alleles have a gene deletion or polymorphisms which lead to no protein expression or the expression of protein without CYP2D6 activity (poor metabolizer (PM)). A third group of alleles comprises polymorphisms, which reduce enzyme activity – intermediate metabolizer (IM) alleles. Depending on their genotype individuals can be extensive/normal, intermediate or poor metabolizers. Extensive or normal metabolizers have EM/PM or EM/EM genotypes and normal metabolism of CYP2D6 substrates. Intermediate metabolizers have IM/IM or IM/PM genotype and CYP2D6 enzyme activity between that of the extensive and poor metabolizers; and poor metabolizers are people with two PM alleles. There is also another type of alleles – ultrarapid metabolizer alleles, which consist of multiplied alleles with normal activity. They are associated with ultra-rapid metabolism of CYP2D6 substrates [39].

In a study of 80 women with newly diagnosed breast cancer, levels of tamoxifen and endoxifen were measured about 4 months after the initiation of therapy. Patients who were poor metabolizers (PM/PM) had between two and four times lower concentrations of endoxifen than those who were with EM/EM and EM/PM genotypes [39]. This trial suggested a gene-dose dependent effect. On the other hand, there are some SSRIs and SNRIs, often prescribed during chemotherapy with tamoxifen for alleviating its side effects (hot flashes), which are also CYP2D6 inhibitors. Thus, SSRIs and SNRIs may have a negative effect on the efficacy of tamoxifen. In a small prospective study of 12 women with breast cancer, the measured plasma concentrations of endoxifen were decreased by around twofold 4 weeks after the beginning of paroxetine (SSRI) intake, which means that CYP2D6 mediates endoxifen metabolism [39].

The role of CYP2D6 genotype was confirmed in a German clinical study of women with ER+ primary invasive breast cancer, as some of them received adjuvant tamoxifen therapy, while others did not [39]. It was observed that patients who were intermediate or poor metabolizers (with PM/PM, EM/PM, IM/IM or IM/PM genotypes) had shorter relapse-free periods and event-free survivals, compared with those who were normal metabolizers (EM/EM or IM/EM genotypes). In the group that did not take tamoxifen, CYP2D6 genotype could not influence survival, which led to the conclusion that CYP2D6 activity cannot be used as prognostic marker for breast cancer, as it only can estimate the outcome with tamoxifen treatment.

There is a great diversity amongst the studies, conducted so far, which makes them hard to compare. For example, none of these studies give information about the connection between CYP2D6 genotype, endoxifen plasma levels and treatment outcome; the concomitant intake of different medicines that act as CYP2D6 inhibitors (not only SSRIs) should also be taken in consideration in all of the trials. Furthermore, tamoxifen dosage varies between individuals, so it may be an important factor in the treatment of patients who are poor metabolizers. In summary, larger, retrospective trials, comparing different population groups, as well as tamoxifen therapy to alternative therapies are necessary before it became possible to apply CYP2D6 testing into everyday practice.

## Interaction of multiple genes on treatment response

A drug response is a complex trait that involves many proteins. From a biological point of view, it is expected that different metabolic routes compete and that the effect of a polymorphism on a drug metabolism pathway can be altered by other polymorphisms.

Fluoropyrimidine-based chemotherapeutic agents, 5-FU or capecitabine, have remained the mainstay of chemotherapeutic regimens for colorectal and gastrointestinal cancers in both metastatic and adjuvant settings.

In a retrospective study of 85 patients with CRC, who were administered fluoropyrimidine-based treatment and 91.8% of the individuals suffered from toxicities, the effect of DPYD, TYMS and MTHFR polymorphisms was investigated. The following polymorphisms were detected: *DPYD* 85 T > C (29.4% heterozygote mutants, 7.1% homozygote mutants), *DPYD* IVS 14 + 1G > A (1.2% heterozygote mutants, 0% homozygous mutants), *TYMS* 1494 del TTAAAG (38.4% heterozygote mutants, 24.7% homozygote mutants), *MTHFR* 677C > T (43.5% heterozygote mutants, 9.4% homozygote mutants) and *MTHFR* 1298A > C (8.2% heterozygote mutants, 2.4% homozygote mutants). In individuals who were heterozygote or homozygote for the MTHFR 1298A > C polymorphism there was an increased frequency of hematopoietic toxicity. No statistically significant association was found between DPYD, TYMS polymorphisms and fluoropyramidine-driven toxicities. Authors suggest that *MTHFR* polymorphisms may be regarded as related factors of fluoropyrimidine toxicity and can be used as predictive biomarkers for patients with CRC who can receive the greatest benefit from fluoropyrimidine-based regimens [40].

In another retrospective study of 108 Chinese patients with metastatic gastric cancer authors assessed the impact of a panel of nine genetic polymorphisms in genes involved in DNA repair (*ERCC1* /rs2298881/, *ERCC2* /rs13181 and rs1799793/ and *XRCC1* /rs25487 and rs25489/), detoxification of oxaliplatin (GSTP1 /rs1695/ and GSTT1 /rs2266637/), and fluoropyrimidine metabolism (MTHFR /rs1801133 and rs1801131/) on predicting clinical response and survival of patients when receiving epirubicin, oxaliplatin and fluorouracil treatment. They found that three of the SNPs had unfavourable effect on the clinical response and survival: *XRCC1* rs25489, homozygous for the A allele; *ERCC2* rs1799793 GA genotype and *ERCC2* rs13181 – heterozygous and homozygous for the G allele. The good-risk group (with no unfavourable SNPs) showed median PFS of 206 days and OS of 534 days, while the poor-risk group (with ≥1 unfavorable SNPs) had PFS of 123 days and OS of 281 days. Thus, the poor-risk group had 2.3-fold increased risk of progression compared with the group with good risk. Based on the results, authors suggest that the following polymorphisms *XRCC1* rs25487, *ERCC2* rs13181 and rs1799793 can be detected before treatment, so that they can be used as a tool for evaluating treatment options in patients with metastatic gastric cancer [41].

## Specific considerations for pharmacogenomics application in oncology practice

One of the most practical application of pharmacogenomics, if sufficient and credible data from multiple clinical trials is collected in the future, would be for the most appropriate choice to be made in disease settings when several equivalent regimens exist. Thus, it would be easier for physicians to select one therapy over another, if the risk of toxicity is high, or alternately, to choose the best regimen, if the expected probability of response is higher. In a situation, in which there is only one option for treatment, sufficient information about the drugs could be of great importance in order toxicity risks to be weighed against potential benefits. On the other hand, if it is well known that potential toxicity risks exist, then the dosage of the chemotherapeutics could be adjusted according to each patient’s genetic profile. Another question that arises is: if we reduce the dose in order to avoid toxicity, will we still have the desirable effect of treatment? This answer can be given only from future large retrospective analyses on drug-gene pairs, if genotyping becomes more common in everyday practice. In the future, most probably clinicians will have to cautiously evaluate the risk-benefit ratio and the pharmacogenomics probability for a certain drug in order to ensure that dose reduction or drug avoidance for reducing side effects or preventing toxicity would not affect the treatment outcome.

Pharmacogenomics is the application of genomic and other “omic” information to help guide, inform and individualize drug therapy. The science underlying pharmacogenetics has evolved rapidly over 50 years since it was first suggested that genetics might influence drug response phenotypes. That process has occurred in parallel with advances in DNA sequencing and other molecular technologies. Pharmacogenomic information is increasingly being integrated into electronic health records (EHRs) and that information is rapidly becoming an important component of the “therapeutic encounter”. The rapid growth of clinically relevant pharmacogenomic knowledge serves to highlight the challenges associated with pharmacogenomic implementation, one of which is that of making this information available to practitioners in a practical and easily understood fashion. To do that requires objective, evidence-based guidelines and investment in the infrastructure required to make pharmacogenomic information accessible to physicians in a timely fashion. As physicians write prescriptions for drugs, not genes, most institutions have focused on drug-gene pairs. To assist care-givers, many institutions have created automatic computer-based alerts that “fire” whenever a drug is prescribed for which a pharmacogenomic test might provide helpful information. For example, at the Mayo Clinic there are 17 drug-gene pairs for which alerts “fire” when being prescribed. These pairs are: Citalopram, Escitalopram, Clopidogrel – CYP2C19; Codeine, Fluoxetine, Fluvoxamine, Paroxetine, Tamoxifen, Tramadol, Venlafaxine – CYP2D6; Triopurines – TMPT; Simvastatin – SLCO1B1; Tacrolimus – CYP3A5; Abacavir – HLA-B*57:01; Allopurinol – HLA-B*58:01; Carbamazapine – HLA-B*15:02 and HLA-A*31:01; Warfarin – CYP2C9 and VKORC1. Decision making with regard to the implementation of an alert depends on evidence-based guidelines that come from sources such as the Clinical Pharmacogenetics Implementation Consortium. Current drug-gene pair alerts are primarily “reactive”, which means that they require that the physician—on the basis of their goals for the patient—order the genetic test in response to the alert. Although an important first step, reactive alerts represent only one step toward the eventual goal, which would involve having pharmacogenomic data for a specific patient “preemptively” available in the electronic health records (EHRs) so there will be no delay associated with waiting for a test result so the pharmacogenomic information can be incorporated into the clinical workflow seamlessly [42].

## Conclusions

The most significant problems that cancer chemotherapy encounters are the development of drug resistance and severe side effects. As most anticancer agents are not tumour specific, they also lead to damage of the normal cells. This prevents the usage of high doses of the drugs, that could be necessary for eradication of the less sensitive populations of tumour cells. The variability in the therapeutic response can be explained by the individual genetic variations, specific for each person. Pharmacogenetic progress can be the keystone to revolutionize cancer therapy. Introducing patient genotyping into clinical settings can facilitate decision making regarding chemotherapy regimens and drug dosages with maximal effect and minimal risk of toxicity. Although many different studies have been conducted so far, still more information is necessary in order personalized medicine to be applied into everyday practice.

Recent studies prove that pharmacogenetics is a promising tool for personalised medicine.

## Data Availability

All data generated or analysed during this study are included in this published article.

## Abbreviations

5-FU: 5-fluorouracil
6-MP: 6-mercaptopurine
ABC: ATP-binding Cassette
ADR: Adverse drug reaction
ATP: Adenosine Triphosphate
CRC: Colorectal carcinoma
CYP2D6: Cytochrome P450 2D6
DNA: Deoxyribonucleic Acid
DPD: Dihydropyramidine dehydrogenase (enzyme)
DPYD: Dihydropyramidine dehydrogenase (gene)
EM: Extensive metabolizer
GST: Glutathione S-transferase
EHRs: Electronic health records
EOF: Epirubicin, Oxaliplatin, Fluorouracil
ERCC1: Excision repair cross complementing group 1
ERCC2: Excision repair cross complementing group 2
IBD: Inflammatory bowel disease
IHC: Immunohistochemistry
IM: Intermediate metabolizer
MDR1: Multi drug resistance
MTHFR: Methylene tetrahydrofolate reductase
MTX: Methotrexate
NSCLC: Non-small cell lung cancer
OS: Overall survival
PD: Progression Disease
PFS: Progression-free survival
PM: Poor metabolizer
SNP: Single nucleotide polymorphism
TG: Thioguanine
TGN: Thioguanine Nucleotide
TPMT: Thiopurine S-methyltransferase
TS: Thymidylate synthetase (enzyme)
TYMS: Thymidylate synthetase (gene)
UGT: Uridine diphosphate glucuronosyl transferase
XRCC1: X-ray cross complementing group 1
